# Primary Treatment Response Rather than Front Line Stem Cell Transplantation Is Crucial for Long Term Outcome of Peripheral T-Cell Lymphomas

**DOI:** 10.1371/journal.pone.0121822

**Published:** 2015-03-27

**Authors:** Giuseppe Gritti, Cristina Boschini, Andrea Rossi, Federica Delaini, Anna Grassi, Alessandra Algarotti, Caterina Micò, Rosangela Trezzi, Andrea Gianatti, Anna Maria Barbui, Alessandro Rambaldi

**Affiliations:** 1 Hematology and Bone Marrow Transplant Units, Azienda Ospedaliera Papa Giovanni XXIII, Bergamo, Italy; 2 Pathology Unit, Azienda Ospedaliera Papa Giovanni XXIII, Bergamo, Italy; European Institute of Oncology, ITALY

## Abstract

Outcome of systemic peripheral T-cell lymphomas (PTCL) is unsatisfactory and no controlled clinical study guides the therapy. Phase II studies suggest to consolidate response achieved after front-line treatment with stem cell transplant (SCT). We retrospectively evaluate the impact of front-line SCT consolidation in a single Center cohort of 209 patients treated during the last two decades. Median age was 49 years (range 15-85) with a prevalence of male sex (61%), advanced stage (68%) while IPI was >2 in 44%. Primary treatment was MACOP-B (39%) CHO(E)P (39%), intensive regimens (18%) or others (4%). Complete response to primary treatment (i.e. before SCT) was 60% (5% partial remission). Forty-four patients further proceeded to SCT while 92 did not receive consolidation. Outcome of primary responders was good, with a 3-year overall survival of 74% (82% in ALCL ALK+ and 69% for the other histologies). By multivariate analysis a better overall survival was significantly associated with IPI<2 (P=0.001), primary response (P=0.000), and ALCL ALK+ (P=0.012). The multivariate analysis performed on responders, showed that only IPI was predictive of a better survival while ALCL ALK+ and undergoing SCT were not. Response to primary treatment rather than post-remission programs is the crucial determinant of PTCL outcome.

## Introduction

Systemic peripheral T-cell lymphomas (PTCL) are a rare and heterogeneous group of non-Hodgkin lymphomas characterized by an aggressive clinical course and poor treatment response.

Several retrospective studies have shown that long-term survival of most PTCL do not exceed 30–40%, with systemic anaplastic large cell lymphoma (ALCL) expressing the anaplastic large cell lymphoma kinase (ALK) protein being the exception[1–4]. The optimal treatment of these lymphomas is still a matter of debate as conventional therapy based on CHOP/CHOP-like regimens provided unsatisfactory outcome[5, 6]. Attempts to improve response and survival with early treatment intensification led to similarly disappointing results[7–11]. Several retrospective studies [12–15] and few small-sized phase II trials[16–20] suggested that consolidation of the first response with high dose therapy (HDT) followed by autologous stem cell transplant (SCT) is a feasible option and may guarantee better disease control, with long-term overall survival (OS) up to 60–70%. This concept has been tested in a large phase II trial enrolling 160 histologically proved patients that reported an encouraging 5-year OS and progression free survival (PFS) as good as 51% and 44%, respectively[21]. Despite these interesting results, no comparative trial is available and it is not clear whether HDT plus autologous SCT should be considered a standard treatment in all PTCL subgroups. In addition, the role of allogeneic SCT as front-line treatment for these patients have been recently reported in a phase II clinical study[22].

In this study, we retrospectively evaluated the outcome of PTCL patients treated in the last two decades at our Center with the aim to evaluate the role of post-remission consolidation with SCT.

## Subjects and Methods

### Patients

Eligible for this retrospective analysis were untreated patients with systemic PTCL diagnosed between January 1990 and December 2012 at “*Papa Giovanni XXIII*” Hospital (*formerly “Ospedali Riuniti*”) of Bergamo. Patients treated with a palliative intent, those without pathological material suitable for revision and primary cutaneous T-cell lymphomas were excluded from the study. The diagnostic slides were independently reviewed by two hematopathologists and classified according to the 2008 WHO classification[23]. When necessary, immunostains were performed i.e. ALK in ALCL. Clinical information was gathered from the electronic charts. When necessary, the paper charts were reviewed. Patient’s written informed consent for the retrospective use of clinical data gathered from the electronic charts was obtained in all cases and this procedure was approved from Hospital’s Ethic Committee (“*Comitato Etico della Provincia di Bergamo*”). The study was approved and conducted in accordance with the Italian laws and the Declaration of Helsinki.

### Treatments and outcome evaluation

Treatments included standard CHOP (cyclophosphamide, doxorubicin, vincristine, and prednisone), CHOP with etoposide (CHOEP), MACOP-B[24] or intensive therapy including both clinical trials[16, 22] and intensive acute lymphoblastic leukemia-like schemes[25]. Patients with stage II–IV PTCL (excluding ALCL ALK positive after 2003), age <60 years with no medical contraindications were considered eligible to first-line SCT consolidation if a clinical response is achieved after a full course of treatment. Patients proceeding to autologous SCT received an additional stem cell mobilizing cycle (MAD or high-dose cyclophosphamide) followed by HDT according to BEAM (BCNU, etoposide, cytosine arabinoside, and melphalan) plus autologous SCT. Front-line allogeneic SCT was performed within a clinical trial[22]. Treatment response was defined according to the 1999 International Working Group criteria[26] and was recorded after the primary treatment (i.e. CHOP, CHOEP, MACOP-B or intensive therapy) and after SCT consolidation when applicable.

### Statistical analysis

The long-term outcome was assessed in terms of overall survival (OS), progression-free survival (PFS) and disease-free survival (DFS) defined according to Cheson criteria[26]. The OS was defined as the time from the start of treatment to death for any cause. The PFS was defined as the time from the start of treatment to disease progression or death for any cause. The DFS was defined as the time from documentation of complete response to relapse or death for any cause. All the variables were analyzed by the Kaplan-Meier method. Patients were censored at the date of last contact, follow-up was updated in September 2013 and all living patients had been observed at least once in the previous 3 months. Differences in survival between groups were identified by generalized log-rank analysis. Multivariate analysis for survival was performed by Cox regression model; proportional hazards assumption was tested. A chi-squared test for independence was used to compare patient’s characteristics according to SCT consolidation.

## Results

### Clinical characteristics

Two-hundred and nine patients were included in the retrospective analysis. The baseline clinical characteristics are summarized in Table 1. Median age at diagnosis was 49 years with a male predominance (61%). In the majority of the cases (68%), patients had an advanced stage disease with an intermediate-high or high International Prognostic Index (IPI)[27] in 44%. There was a prevalence of ALCL (51%), particularly ALK positive (34%). These latter patients were characterized by lower median age than other histologies (42.6 vs 57.1 years, P = <0.0001) and showed lower IPI and PIT scores (P = 0.0017 and 0.0392, respectively). Primary treatment was MACOP-B in most of the cases until 2003 and CHOP or CHOEP later on while 18% of the patients received intensive treatments (Table 2).

**Table 1:** Patients Characteristics.

	All N (%)	Non-ALCL ALK+ N (%)	ALCL ALK+ N (%)	P
All patients	209 (100)	139 (66)	70 (34)	
Age				
*Median (range)*	49.3 (15–85)	57.1 (19–85)	42.6 (15–82)	<0.0001
≥*60*	69 (33)	60 (43)	9 (13)	<0.0001
Gender				
*Male*	128 (61)	80 (58)	48 (69)	0.1228
Histology				
*PTCL-NOS*	67 (32)	67 (48)		
*ALCL ALK positive*	70 (34)	-	70 (100)	
*ALCL ALK negative*	36 (17)	36 (26)	-	
*AITL*	21 (10)	21 (15)	-	
*EATL*	10 (5)	10 (7)	-	
*Others*	5 (2)	5 (4)	-	
Ann Arbor Stage				
*III–IV*	143 (68)	102 (73)	41 (59)	0.0297
ECOG PS				
≥*2*	87 (42)	54 (39)	33 (47)	0.2464
Serum LDH Level				
*> ULN*	92 (44)	64 (46)	28 (40)	0.3585
Extranodal Sites				
*>1*	68 (33)	54 (39)	14 (20)	0.0057
Bone marrow involvement				
*Yes*	22 (12)*	18 (15)**	4 (7)***	0.1153
IPI				
*>2*	91 (44)	71 (51)	20 (29)	0.0017
PIT				
*>2*	26 (15)*	22 (18)**	4 (7)***	0.0392

Legend:

* N = 179;

** N = 120;

*** N = 59;

*PTCL-NOS*: *Peripheral T-Cell Lymphoma Not Otherwise Specified; ALCL*: *Anaplastic Large-Cell Lymphoma; ALK*: *Anaplastic Large Cell Lymphoma Kinase; EATL*: *Enteropathy-Associated T-cell Lymphoma; AITL*: *Angioimmunoblastic T-cell Lymphoma; Others*: *includes hepatosplenic T-cell lymphoma (N = 3) and extranodal T/NK-cell lymphoma nasal type (N = 2);IPI*: *International Prognostic Index; PIT*: *Prognostic Index for PTCL-NOS*

**Table 2:** Treatments and Outcome.

	All N (%)	Non-ALCL ALK+ N (%)	ALCL ALK+ N (%)	P
All patients	209 (100)	139 (67)	70 (33)	
Primary treatment				
*CHO(E)P*	82 (39)	65 (47)	17 (24)	0.0001
*MACOP-B*	81 (39)	39 (28)	42 (60)	
*Intensive regimens*	37 (18)	29 (21)	8 (12)	
*Others*	9 (4)	6 (4)	3 (4)	
Response to primary treatment				
*CR*	126 (60)	79 (57)	47 (67)	0.1300
*PR*	10 (5)	6 (4)	4 (6)	
*SD/PD*	45 (22)	30 (22)	15 (21)	
*Early death*	28 (13)	24 (17)	4 (6)	

Legend: *Others*: *includes MetAspDex regimen*, *ACVBP or ACOD; CR*: *Complete Remission; PR*: *Partial Remission; SD*: *Stable Disease; PD*: *Progressive Disease; SCT*: *Stem Cell Transplant*. *Early death indicates patients deceasing <6 months from diagnosis*.

### Response and survival in the whole cohort

Overall response rate to primary treatment (i.e. before SCT consolidation) was 65% with a complete response (CR) achieved in 60% of patients (Table 2). Of note, 13% of the patients died early during treatment (<6 months), with a similar incidence among the three treatment categories. Treatment response was slightly better in ALCL ALK positive patients than the others, however the result was not statistically significant (P = 0.130). Consolidation of first response with SCT was performed in 44 patients (21%). In most of these cases (N = 41/44, 93%) the stem cell source was autologous while an allogeneic SCT was performed only in 3 cases. Six of the seven patients undergoing autologous SCT in partial remission (PR) achieved the CR. Of the three patients undergoing allogeneic SCT, one deceased by pulmonary aspergillosis shortly after engraftment, one deceased by grade IV cutaneous and intestinal acute graft versus host disease (GVHD) at day 64 and the last patient is alive and well after nearly 5 years.

**Fig 1:**
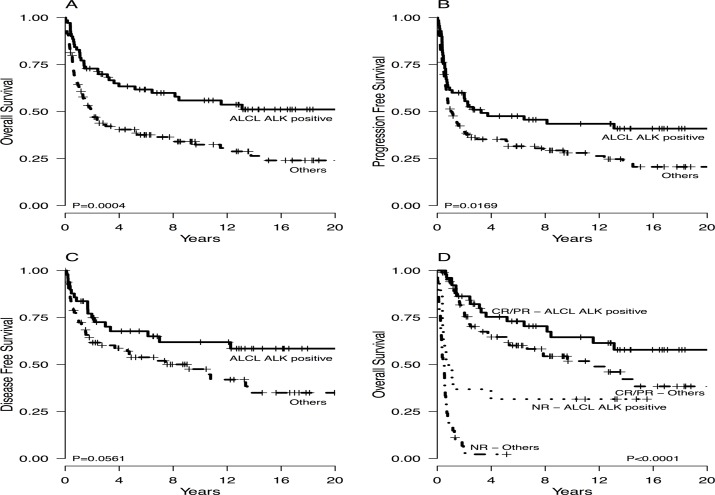
Overall survival (A), progression-free survival (B) and disease free survival (C) in the whole cohort according to histologic subgroup. Overall survival according to primary treatment response (E). ALCL: anaplastic large cell lymphoma; ALK: anaplastic large cell lymphoma kinase; CR: complete remission; PR: partial remission; NR: no response, including stable and progressive disease.

Overall survival, PFS and DFS of patients with ALCL ALK positive versus other histologies are presented in Fig. 1, while the impact of the different histology on clinical outcome is shown in S1 Fig.

It was not possible to evaluate the impact of specific histology on outcome due to the relatively small sample size. Interestingly, the 5 patients with Enteropathy-Associated T-cell Lymphoma (EATL) who performed autologous SCT remained all disease free after a median follow-up of 5.61 (0.63–7.93) years.

By univariate analysis, an inferior clinical outcome was related to IPI >2 [Hazard Ratio (HR) 2.90, P<0.001], Prognostic Index for PTCL-unspecified (PIT)[28] >2 (HR 3.29, P<0.001), the male sex (HR 1.60, P = 0.016), the lack of a clinical response to primary treatment (HR 6.61, P<0.001), PTCL not otherwise specified (NOS) histology compared with ALCL ALK positive (HR 2.54, P<0.001) and MACOP-B treatment (HR 0.59, P = 0.040) while year of treatment >2003 (HR 1.32, P = 0.153) did not show any impact. By multivariate analysis IPI, ALCL ALK positive histology and primary treatment response were independent predictors of outcome (Table 3).

**Table 3:** Multivariate analysis for overall survival of the whole cohort.

	Hazard Ratio	CI 95%	P	3-year OS	10-year OS
Age					
*< 60*	1.00			59%	47%
≥*60*	1.20	0.77–1.88	0.4251	38%	26%
IPI					
≤ *2*	1.00			68%	56%
*> 2*	1.82	1.21–2.74	0.0041	32%	21%
Primary treatment response					
*NR/EXITUS*	1.00			12%	11%
*CR/PR*	0.14	0.09–0.22	<0.0001	74%	57%
Histology					
*ALCL ALK positive*	1.00			70%	56%
*Others*	2.78	1.68–4.59	<0.0001	43%	33%
Chemotherapy scheme					
*CHO(E)P*	1.00			42%	28%
*MACOP-B*	1.00	0.57–1.76	0.9998	62%	50%
*Intensified*	0.78	0.46–1.34	0.3760	51%	45%
Year of treatment					
≤ *2003*	1.00			57%	44%
*> 2003*	0.77	0.48–1.25	0.2906	44%	-

### Response and survival according to primary treatment response

Overall survival according to primary treatment response shows that patients responding (CR/PR) to primary treatment fares well, with a global 3-year OS of 74% that was 82% in ALCL ALK positive patients and 69% for the other histologies (Fig. 1D). We next analyzed the effect of consolidation with autologous SCT in our cohort of responsive patients. For this purpose, we excluded the three patients receiving allogeneic SCT, and analyzed our patient population in two different cohort according to histology i.e. ALCL ALK positive and others. With the exception of age, the presenting clinical characteristic of non-ALCL ALK positive patient who received or not an autologous SCT after achieving a clinical response did not significantly differ (Table 4).

**Table 4:** Clinical Characteristics of Patients Responsive to Primary Treatment.

	Non-ALCL ALK+			ALCL ALK+		
	With SCT N (%)	Without SCT N (%)	P	With SCT N (%)	Without SCT N (%)	P
All patients	26 (32)	56 (68)		15 (29)	36 (71)	
Age						
*Median (range)*	45.3 (19–62)	63.9 (22–85)	0.0002	31.4 (20–59)	44.1 (17–79)	0.0265
≥*60*	2 (8)	32 (57)	<0.0001	0 (0)	5 (14)	0.1286
Sex						
*Male*	16 (62)	29 (52)	0.4089	9 (60)	24 (67)	0.6499
Histology						
*PTCL-NOS*	7 (27)	28 (50)	0.0209	0	0	-
*ALCL ALK positive*	0	0		15 (100)	36 (100)	
*ALCL ALK negative*	8 (31)	15 (27)		0	0	
*AITL*	4 (15)	11 (20)		0	0	
*EATL*	5 (19)	1 (1.5)		0	0	
*Others*	2 (8)	1 (1.5)		0	0	
Ann Arbor Stage						
*III–IV*	14 (54)	39 (70)	0.1638	12 (80)	16 (44)	0.0201
IPI						
*>2*	6 (23)	23 (41)	0.1127	6 (40)	6 (17)	0.0735
PIT						
*>2*	2 (9)	3 (6)	0.6185	2 (13)	0 (0)	0.0519
Relapse/progression						
*Yes*	9 (35)	22 (39)	0.6849	2 (13)	13 (36)	0.1038
Primary treatment response						
*CR*	21 (81)	55 (98)	0.0048	13 (87)	34 (94)	0.3465
*PR*	5 (19)	1 (2)		2 (13)	2 (6)	
Final response (after autologous SCT)						
*CR*	25 (96)	-	-	15 (100)	-	-
*PR*	1 (4)	-	-	0	-	-

*Legend*: *PTCL-NOS*: *Peripheral T-Cell Lymphoma Not Otherwise Specified; ALCL*: *Anaplastic Large-Cell Lymphoma; ALK*: *Anaplastic Large Cell Lymphoma Kinase; EATL*: *Enteropathy-Associated T-cell Lymphoma; AITL*: *Angioimmunoblastic T-cell Lymphoma; Others*: *includes hepatosplenic T-cell lymphoma (N = 3) and extranodal T/NK-cell lymphoma nasal type (N = 2); SCT*: *Stem Cell Transplant*

Conversely, transplanted patients with ALCL ALK positive were at higher risk according to stage, PIT, and probably IPI. Of note, autologous SCT converted a PR in CR in 6 out of 7 cases. By multivariate analysis, only IPI >2 was associated with an inferior survival (HR 2.37, P = 0.018), while the clinical benefit related to SCT consolidation and ALCL ALK positive histology was not confirmed (Table 5). When excluding ALCL ALK positive patients from the analysis, the results did not change and receiving SCT was not associated to better prognosis (Table 6).

**Table 5:** Multivariate analysis for overall survival of the responders*.

Variables	Hazard Ratio	CI 95%	P	3-year OS	10-year OS
Age					
*< 60*	1.00			79%	61%
≥*60*	1.22	0.58–2.58	0.6013	63%	47%
IPI					
≤ *2*	1.00			80%	64%
*> 2*	2.14	1.14–4.02	0.0174	64%	41%
1^st^ line autologous SCT consolidation					
*Performed*	0.66	0.33–1.33	0.2443	82%	65%
Histology					
*ALCL ALK positive*	1.00			82%	64%
*Others*	1.65	0.88–3.10	0.1208	70%	41%
Chemotherapy scheme					
*CHO(E)P*	1.00			69%	46%
*MACOP-B*	1.18	0.60–2.31	0.6300	74%	58%
*Intensified*	0.69	0.25–1.93	0.4819	88%	82%

*Legend*:

* excluding 3 patients treated with allogeneic SCT

**Table 6:** Multivariate analysis for overall survival of the responders* (excluding ALCL ALK+).

Variables	Hazard Ratio	CI 95%	P	3-year OS	10-year OS
Age					
*< 60*	1.00			79%	61%
≥*60*	1.59	0.70–3.61	0.2685	57%	37%
IPI					
≤ *2*	1.00			78%	66%
*> 2*	2.65	1.29–5.43	0.0077	55%	25%
1^st^ line autologous SCT consolidation					
*Performed*	0.97	0.41–2.27	0.9436	80%	53%
Chemotherapy scheme					
*CHO(E)P*	1.00			62%	38%
*MACOP-B*	0.77	0.35–1.72	0.5311	73%	56%
*Intensified*	0.68	0.24–1.93	0.4702	85%	76%

*Legend*:

* excluding 3 patients treated with allogeneic SCT

The clinical outcome of the 45 patients not responding to primary treatment was remarkably poor, with a 1-year OS of 19% (Fig. 1D) that was as low as 4% when excluding ALCL ALK positive patients. For these patients a salvage autologous SCT was performed in 7 cases (16%) and an allogeneic SCT in 6 (13%). Long-term survivors were only 6 patients (all with a diagnosis of ALCL ALK positive) who received either an allogeneic (N = 3) or autologous (N = 3) SCT. On the contrary, outcome of patients treated with chemotherapy only was very poor, with only one long-term survivor that, however, experienced multiple chemosensitive relapse.

Twenty-eight patients (13%) died early during treatment. The median age of this latter cohort of patients was 62.0 years (range 35–84), 21 patients (75%) showed an IPI >2 and the disease was symptomatic in 20 cases (71%). In most of the cases (89%) cause of death was infection, in large part associated to evidence of insufficient disease control.

## Discussion

Over the last two decades, consolidation of first-response with HDT and autologous SCT has been explored in systemic PTCL with the intent to reduce the unacceptable high relapse rate of this group of lymphomas. The heterogeneity and relative rarity of these diseases was a great limit to perform informative trials and only few, small sized non-comparative prospective studies have been available until recently. Several studies have shown a long-term (≥3 years) OS ranging from 48 to 73% after HDT and autologous SCT[15–18, 20]. Recently, a large phase II trial including 160 histologically proven PTCL, with the exclusion of ALCL ALK positive, reported encouraging survival data, with a 5-year OS and PFS of 51% and 44%, using a dose dense CHOEP-14 induction therapy (CHOP-14 if age >60 years) and autologous SCT in responders[21]. More recently, allogeneic SCT has been as well explored in first-line setting[22].

In this study, we present the outcome of a large cohort of histologically confirmed PTCL patients derived from a single Institution and followed up for a prolonged period of time. To our knowledge, this is the first report that, though retrospectively, describes the outcome of responding patients according to subsequent consolidation strategy. Our results indicate that response to primary treatment is the key variable for the long term prognosis and consolidation with HDT and SCT may not be crucially important for most of the complete responders. However, in some patients such as those with EATL, first-line SCT looked particularly beneficial when performed in CR. Although not statistically significant due to the limited sample size, these data are in line with other recently published.[29] In addition, in some patients we could observe the ability of SCT consolidation in converting a partial response into a complete one.

Median age of our cohort was lower and the proportion of ALCL ALK positive patients was higher compared to other series.[4] This finding may reflect the pattern of referral as our Center is the tertiary care referral center, and is accordingly similar to those reported in other report, for example in a German series of 320 patients enrolled in clinical trials median age was 50 years and 60% of the patients had a diagnosis of ALCL (41% ALK positive)[9]. We included in the current analysis patients with ALK positive ALCL, that are not usually considered eligible for consolidation therapy, despite the severe prognosis of high risk disease[3]. Reason for this choice were the homogeneous good prognosis across histologies in responding patients, confirmed by multivariate analysis. Additionally, we could confirm that the results of the study did not change excluding ALCL ALK positive patients.

The role of allogeneic transplantation as a front line consolidation treatment option could not be addressed by this study and is the matter of investigation of a different ad hoc designed clinical trial. Therefore, while waiting for additional data, allogeneic SCT consolidation should be used with caution in patients responding to a first line treatment.

The primary determinant of outcome resulted to be treatment response, and despite several clinical trials have been addressing the unsatisfactory outcome of PTCL in the last two decades, 6 to 8 cycles of chemotherapy with CHOP with or without etoposide (CHOEP) still remains the standard[30, 31]. Several novel molecules are currently facing the clinical ground and there is high expectation from the combination of standard chemotherapy with newer biological agents[32]. Despite the rapid growth of clinical trials in PTCL driven by new drugs, the role of consolidation with HDT and autologous SCT remains not addressed, yet.

Conversely, since primary response is such a key factor for outcome, there is an urgent need to develop strategies aimed at the early identification of poor responders. In this respect, the role of interim ^18^F-fluorodeoxyglucose—positron emission tomography (FDG—PET) remains controversial but in general, differently from Hodgkin disease[33], it does not provide informative data as to chemosensitivity of this group of patients[34–36].

In conclusion, our study underlines the crucial importance of increasing the quality of response to primary treatment and the need of new drugs or innovative treatment strategies to achieve such a goal. Among these latter treatment strategies, the role of a consolidation of first remissions with autologous stem cell transplantation should always be considered[37] but its benefit remains to be proven by well conducted comparative, prospective, clinical trials.

## Supporting Information

S1 Fig Overall survival (A) and disease-free survival (B) according to histology in the whole cohort. PTCL-NOS: Peripheral T-Cell Lymphoma Not Otherwise Specified; ALCL: Anaplastic Large-Cell Lymphoma; ALK: Anaplastic Large Cell Lymphoma Kinase; EATL: Enteropathy-Associated T-cell Lymphoma; AITL: Angioimmunoblastic T-cell Lymphoma; Others: includes hepatosplenic T-cell lymphoma and extranodal T/NK-cell lymphoma nasal type.
(TIFF)
