# Supplementary figures and images for: Primary Treatment Response Rather than Front Line Stem Cell Transplantation Is Crucial for Long Term Outcome of Peripheral T-Cell Lymphomas
Source: PLoS One. 2015 Mar 27;10(3):e0121822. doi: 10.1371/journal.pone.0121822 (PMC4376730; doi:10.1371/journal.pone.0121822)

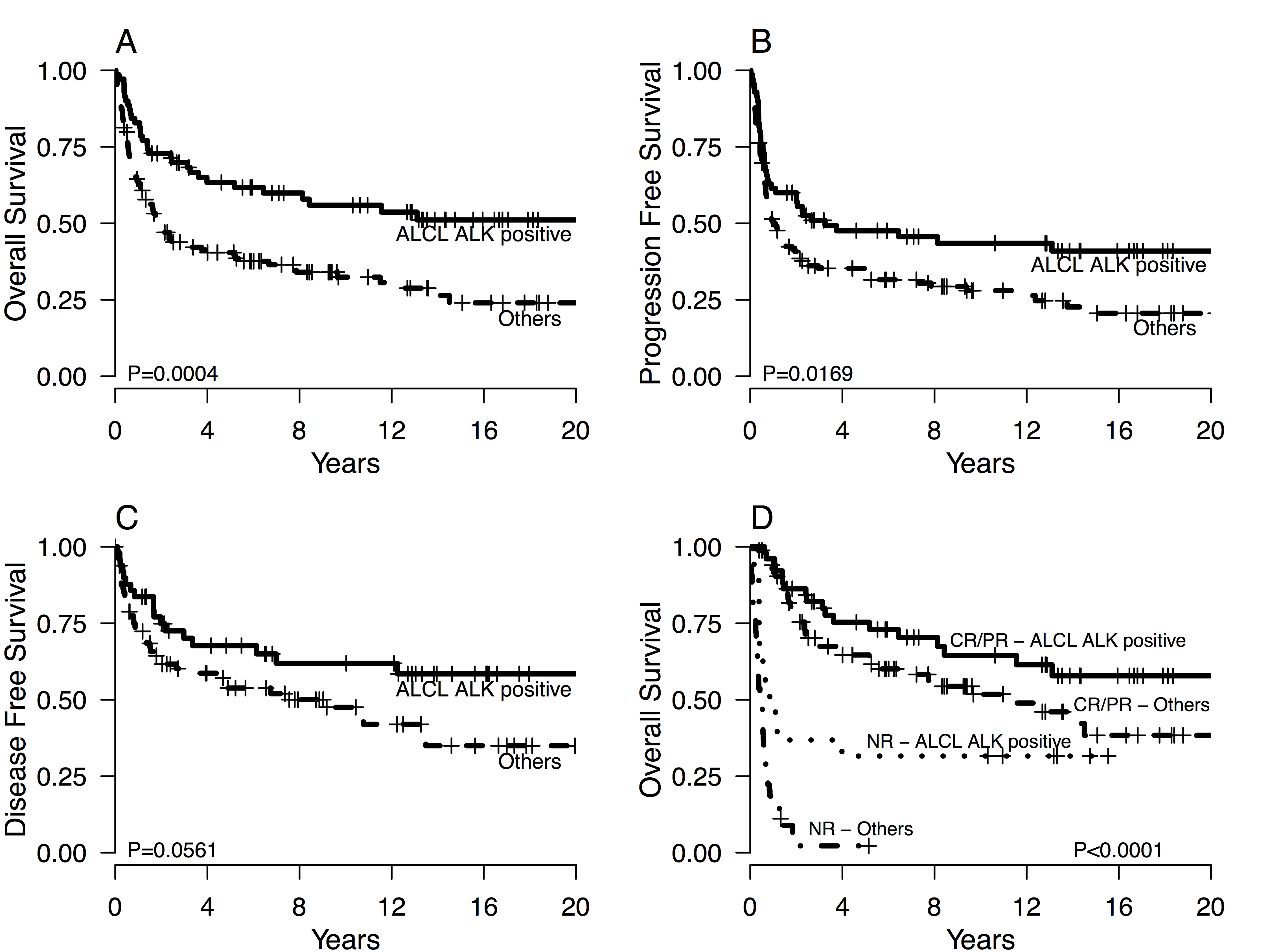

Supplement: S1 Fig — PTCL-NOS: Peripheral T-Cell Lymphoma Not Otherwise Specified; ALCL: Anaplastic Large-Cell Lymphoma; ALK: Anaplastic Large Cell Lymphoma Kinase; EATL: Enteropathy-Associated T-cell Lymphoma; AITL: Angioimmunoblastic T-cell Lymphoma; Others: includes hepatosplenic T-cell lymphoma and extranodal T/NK-cell lymphoma nasal type. (TIFF) [file pone.0121822.s001.tiff]
